# Ultrasound-Assisted Hydrazine Reduction Method for the Preparation of Nickel Nanoparticles, Physicochemical Characterization and Catalytic Application in Suzuki-Miyaura Cross-Coupling Reaction

**DOI:** 10.3390/nano10040632

**Published:** 2020-03-28

**Authors:** Adél Anna Ádám, Márton Szabados, Gábor Varga, Ádám Papp, Katalin Musza, Zoltán Kónya, Ákos Kukovecz, Pál Sipos, István Pálinkó

**Affiliations:** 1 Department of Organic Chemistry, University of Szeged, Dóm tér 8, H-6720 Szeged, Hungary; lee-daa@hotmail.com (A.A.Á.); szabados12m@gmail.com (M.S.); gaborvarga1988@gmail.com (G.V.); musza.katalin@chem.u-szeged.hu (K.M.); 2 Material and Solution Structure Research Group, and Interdisciplinary Excellence Centre, Institute of Chemistry, University of Szeged, Aradi Vértanúk tere 1, H-6720 Szeged, Hungary; papy97@gmail.com (Á.P.); sipos@chem.u-szeged.hu (P.S.); 3 Department of Applied and Environmental Chemistry, University of Szeged, Rerrich B. tér 1, H-6720 Szeged, Hungary; konya@chem.u-szeged.hu (Z.K.); kakos@chem.u-szeged.hu (Á.K.); 4 MTA-SZTE Reaction Kinetics and Surface Chemistry Research Group, Rerrich B tér 1, H-6720 Szeged, Hungary; 5 Department of Inorganic and Analytical Chemistry, University of Szeged, Dóm tér 7, H-6720 Szeged, Hungary

**Keywords:** nickel nanoparticles, sonocrystallization, structural and morphology characterization, acid-base properties, activities in the Suzuki-Miyaura cross-coupling reaction

## Abstract

In the experimental work leading to this contribution, the parameters of the ultrasound treatment (temperature, output power, emission periodicity) were varied to learn about the effects of the sonication on the crystallization of Ni nanoparticles during the hydrazine reduction technique. The solids were studied in detail by X-ray diffractometry, dynamic light scattering, thermogravimetry, specific surface area, pore size analysis, temperature-programmed CO_2_/NH_3_ desorption and scanning electron microscopy. It was found that the thermal behaviour, specific surface area, total pore volume and the acid-base character of the solids were mainly determined by the amount of the nickel hydroxide residues. The highest total acidity was recorded over the solid under low-power (30 W) continuous ultrasonic treatment. The catalytic behaviour of the nanoparticles was tested in a Suzuki-Miyaura cross-coupling reaction over five samples prepared in the conventional as well as the ultrasonic ways. The ultrasonically prepared catalysts usually performed better, and the highest catalytic activity was measured over the nanoparticles prepared under low-power (30 W) continuous sonication.

## 1. Introduction

Nanoparticles (NPs) are commonly defined as solid materials with at least one dimension in the 1–100 nanometer size range including even polycrystalline systems with nano-sized crystallites [1]. Nanoparticles exhibit several interesting size-dependent properties, which are different from their bulk counterparts, such as large surface to volume ratio [2], optical [3,4], magnetic [5,6] and electronic attributes [7]. The dimensions of the nanoparticles are comparable to those of biological moieties like viruses (20–50 nm), proteins (5–50 nm) or genes (2 nm wide and 10–100 nm long) revealing the potential to tag or address these units with NPs. Moreover, some nanoparticles are ferromagnetic, and they can be easily led by an external magnetic field, therefore, their utilization in cancer research or in healing processes as targeted drug delivery system is a research field in the lime-light [8,9]. Their special and tuneable physicochemical properties are connected to wide range of industrial uses (e.g., nano-carbon: fillers and black pigment, titania nanoparticles: UV protection, whitening pigment and solar cell) [10]. The relatively easy control of shape and size of nanoparticles, the enhanced specific surface area compared to the bulk-phase variants make possible to rationally design the materials for catalytic applications [1,11].

As economically friendly alternatives of Pd and Pt, Ni nanoparticles have received significant attention in the field of the catalysis in the last decades. They are frequently utilized in gas-phase reactions [12,13,14], e.g., Vargas et al. used *m*-ZrO_2_ supported Ni nanoparticles as catalyst in CO_2_ methanation, and the experimental results showed excellent catalytic activity and selectivity to CH_4_ formation [12]. There are also several examples for their application in the liquid phase: the Suzuki cross-coupling [15] and the Ullmann coupling [16] reactions are generally used as probe reactions for Ni NPs.

It is clear from previous studies that the controlled synthesis of monodisperse nanoparticles is extremely important for the diverse applications. Fundamentally, there are two different ways for the preparation of nanocrystals: (i) one is the top-down approach, which mainly applies physical methods to reduce the size of the particles from the bulk phase to the nano-range using mechanical energy to prepare nanoparticles via processes connected to deformation, defragmentation, cold welding or even recrystallization [17,18,19,20], (ii) the other is the bottom-up approach, which involves methods usually performing better as far as the synthesis of size- and shape-control of the nanoparticles are concerned.

In 1857, Faraday reported a preparation way for the colloidal gold sol via the reduction of HAuCl_4_ by phosphorous reagent [21]. For chemical reduction, several reducing agents such as hydrazine [22,23], sodium borohydride [24] and alcohols [25] can be easily and efficiently applied. Sol-gel method is a generally used technique to synthesise nanoparticles with desired size and stoichiometric control [26]. Another commonly applied pathway is thermal decomposition, where the solution of the mixed precursors and additives (like surfactants) is heated, and ultimately, the solid residue is calcined and nanoparticles are formed [27]. Finally, it is worthwhile to mention an alternative and green biological synthetic method, where the reducing agents are extracted from plants, for instance from the leaf of *Aloe vera* [28], *Cinnamomum camphora* [29] or *Coriandrum sativum* [30].

Nowadays, much attention is paid to sonochemistry due to the unique effects of the extreme reaction conditions (high temperature and pressure) occurring inside the continuously generated and collapsed acoustic cavities. These voids can efficiently accumulate the energy of ultrasound waves and transform into mechanical, thermal, compressional and even light forms. In addition, part of the sonication energy results in the formation of reactive H_2_ and O_2_ gases, H_2_O_2_ side-product and hydroxyl radicals in water and aqueous solutions [31]. Similarly to the mechanochemical processes, the transient and inhomogeneously distributed hot spots with unusually fast heating and cooling rates (>10^9^ K/s) can induce unique transformations and the formation of crystal defects not seen otherwise [32,33]. The utility of sonochemical synthesis as a synthetic tool resides in its versatility. By varying the reaction conditions, various forms of nanostructured materials can be prepared, including metals [34,35,36,37], bimetals [38,39], oxides [40,41], sulphides [42] and nanostructured supported [43,44] catalysts. To the best of our knowledge, in the literature only few examples for the synthesis of nickel nanoparticles via ultrasound treatment can be found. They all apply surfactants to avoid the formation of large aggregates and to tailor the morphology of the obtained particles. Vargas et al. synthesized nickel nanoparticles with ultrasound assistance from nickel chloride by chemical reduction investigating the effect of the quality of the reducing agents in detail [12]. In another work, the nickel nanoparticles were sonically prepared on polyester fabric. Field emission scanning electron microscopy observation indicated hedgehog-like nickel particles [45]. The formation of similar aristate spherical Ni NPs were also observed, and the influence of the sonication time was extensively scrutinized regarding the average grain size of the crystals. The authors reported about size growth from 5 nm to 65 nm as the ultrasonic-assisted reduction time increased from 10 min to 150 min [46].

Interestingly, the effect of the ultrasound treatment parameters on the generation of Ni NPs like the temperature of the applied medium, the intensity and pulse character of ultrasound emission remained out of scope. Hence, our aim was to map the influence of the ultrasound treatments on the formation of nickel nanoparticles supplementing the hydrazine reduction method in the absence of surfactant, which are generally the source of the inconveniences for the application of NPs owing to their complicated removal from the outer surface. The sonochemically obtained nanoparticles were compared to those prepared in the conventional way, mechanically or without stirring, in order to reveal the direct effect of the ultrasound waves. The NPs were characterized using a wide-range of instrumental techniques, and their catalytic activities were tested in a Suzuki-Miyaura cross-coupling reaction.

## 2. Materials and Methods

### 2.1. Materials

Anhydrous nickel iodide (NiI_2_), anhydrous potassium hydroxide (KOH) pellets, phenylboronic acid, biphenyl, *N*,*N*-dimethylformamide (DMF), dimethyl sulfoxide (DMSO), toluene and absolute ethanol were purchased from VWR International (EU). Iodobenzene, potassium carbonate (K_2_CO_3_) were acquired from Sigma-Aldrich company (Budapest, Hungary) and the hydrazine monohydrate (N_2_H_4_ × H_2_O) (98+%) was ordered form Alfa Aesar (Haverhill, MA, USA). All chemicals were of 99+% purity and no further purification was required.

### 2.2. The Ways of Ni Nanoparticle Syntheses

The NPs were synthesized via the hydrazine reduction method assisted by ultrasound treatment as well. As the first step, the anhydrous nickel iodide (0.17 g) was dissolved directly in absolute ethanol (4 cm^3^) while, in another vessel, the mixture of the potassium hydroxide (0.31 g) and hydrazine monohydrate (0.35 g) were prepared in 1 cm^3^ of absolute ethanol. The nickel iodide solution and the base/reducing solution were mixed, placed into glass centrifuge tubes (5 cm^3^) and irradiated with ultrasound under ambient atmosphere and pressure at varying temperatures and time durations. For comparison, samples were prepared with mechanical stirring (magnetic stirrer at 1000 rpm and power of 20 W) or without stirring. The sonication was performed with a Hielscher ultrasonic homogenizer (UP200Ht, 26 kHz, max. 200 W) (Hielscher Ultrasonics GmbH, Teltow, Germany) in a thermostated double-walled glass vessel. In this set-up, the target temperatures could be maintained within ±2.0 °C. The applied output power and the emission periodicity of the ultrasound application were altered within the range of 30–120 W and 20–100%, respectively. At 100%, the ultrasonic homogenizer worked continuously, otherwise, the sonication time was a fraction of 100%. For instance, at 20%, the sonication period was one fifth of the active time, and the apparatus was shut off in four-fifth of the pulse by using the pulse on/off mode. The sonotrode of the device was 14 mm in diameter, and the average immersion depth was 25–30 mm. The four tubes were fully dipped into the inner volume of the thermostated glass vessel filled with distilled water, and the tubes were placed 20 mm away from the sonotrode. In this system, indirect ultrasonic treatment was performed; the water medium (in the glass vessel) transferred the ultrasound waves from the sonotrode to the centrifuge tubes. The power calibration of the ultrasonic set-up was performed by the generally used calorimetric method (Table 1) in order to follow and compare the energy dissipation of the various modes of sonication (the inner volume of the glass vessel was 550 cm^3^ and the time profiles in function of time were recorded for 30 min of ultrasound treatment) [47,48]. At the end of the syntheses, the nanoparticles were collected on 0.22 micron filters (Sigma-Aldrich, Budapest, Hungary), and were washed with distilled water and ethanol, dried (at 60 °C) and stored under N_2_ atmosphere.

### 2.3. Procedure of the Suzuki-Miyaura Cross-Coupling Reactions

The catalytic test reactions were carried out in a 10 cm^3^ glass reactor immersed into a preheated oil bath. Iodobenzene (1.0 mmol), phenylboronic acid (1.2 mmol), potassium carbonate (2.5 mmol) and Ni NPs (0.15 mmol) were mixed in 4 cm^3^ of various solvents (DMF, DMSO, toluene), and 1 cm^3^ of distilled water was added to increase the solubility of the K_2_CO_3_ base. The reaction was conducted under reflux, and the reaction time was varied from 1 to 24 h. The reaction was followed primarily by thin-layer chromatography (hexane:ethylacetate 19:1), and at the end of the reaction, the solid catalysts were filtered, and the clear liquids were analysed on a Hewlett-Packard 5890 gas chromatograph (Hewlett-Paclard, Budapest, Hungary) equipped with 50 m × 0.2 mm × 0.33 µm HP-FFAP (nitroterephthalic acid modified polyethylene glycol) column and flame ionization detector. The chromatographic peaks were identified using commercial calibration standards.

### 2.4. Structural Characterization by Instrumental Methods

The powder X-ray diffractograms (XRD) in the *θ* = 4–80º range were recorded on a Rigaku Miniflex II instrument (Rigaku, Tokyo, Japan) with 4°/min scan speed using Cu_Kα_ (*λ* = 1.5418 Å) radiation. The assignation of the reflections in the normalized diffractograms were done with the help of the Joint Committee of Powder Diffraction Standards−International Centre for Diffraction Data (JCPDS-ICDD) database. The primary particle sizes of the NPs were estimated from the most intense 111 reflections using the Scherrer equation with 0.9 shape factor.

The Fourier-transform infrared (FT-IR) spectra were recorded on a JASCO FT/IR-4700 spectrophotometer (Jasco, Easton, MD, USA) with 4 cm^−1^ resolution accumulating 256 scans. The spectrometer was equipped with a DTGS detector and a ZnSe ATR accessory. On the normalized curves, the structural features of the solids were evaluated in the 4000–500 cm^−1^ wavenumber range.

A Malvern NanoZS dynamic light scattering (DLS) instrument (Malvern Pananalytical, Marvel, UK) operating with a 4 mW helium-neon laser light source (*λ* = 633 nm) was used to map the heterogeneity in the solvodynamic diameters of the aggregated nanoparticles at room temperature. The measurements were performed in back-scattering mode at 173°, and the nanoparticles were predispersed in ethylene glycol for 1 h using ultrasonic treatment. The concentration of the light-grey semi-transparent dispersion was 0.1 mg/cm^3^.

In order to gain information about the thermal behaviour of the nanoparticles between 25 and 1000 °C, the dried samples were analysed by a Setaram Labsys derivatograph (Setaram Instrumentation, Caluire, France) applying constant air flow at 3 °C/min heating rate. For the measurements, 15–25 mg of the samples were placed into high-purity alpha alumina crucibles.

The N_2_ adsorption-desorption isotherms were registered on a Quantachrome NOVA 3000e instrument (Quantachrome Instruments, Munich, Germany). The nanoparticles were degassed at 200 °C for 3 h in vacuum to remove the surface-adsorbed species. The specific surface areas were calculated by the Brunauer–Emmett–Teller equation from the adsorption branches, and the determination of the average pore sizes and total pore volumes were estimated from the desorption branches by the Barett–Joyner–Halenda equation.

The basic and acid sites of the solids were characterized by temperature-programmed desorption (TPD) using 99.9% CO_2_/He and 99.3% NH_3_/He (50 cm^3^/min flow), respectively. TPDs were performed on a BELCAT-A catalyst analyser (Microtrac MRB, Munich, Germany) equipped with thermal conductivity detector. Before the measurements, about 20 mg of the samples were purified/degassed in He atmosphere and quartz cell at 300 °C for 1 h, then the CO_2_ and NH_3_ saturation were conducted at 40 °C and 90 °C, respectively. The TPD profiles were registered up to 600 °C with 10°C/min heating rate.

The morphology and size of the nanocrystals were examined by scanning electron microscopy (SEM-Hitachi S-4700 instrument) (Hitachi, Tokyo, Japan). For these measurements, the solids were placed onto conductive carbon adhesive tapes, and a few nm of gold-palladium alloy films were sublimed onto the surface of the samples in order to avoid charging. Elemental analysis was performed by energy dispersive X-ray (EDX) measurements (Röntec QX2 spectrometer, Röntec GmbH, Berlin, Germany), equipped with Be window and coupled to the SEM.

## 3. Results and Discussion

The influence of the ultrasonic treatment on the formation of the nanoparticles were studied in detail varying the intensity and the emission character of the ultrasound treatment.

### 3.1. Effects of the Sonication on the Physicochemical Properties of the Nickel Nanoparticles

**Figure 1:**
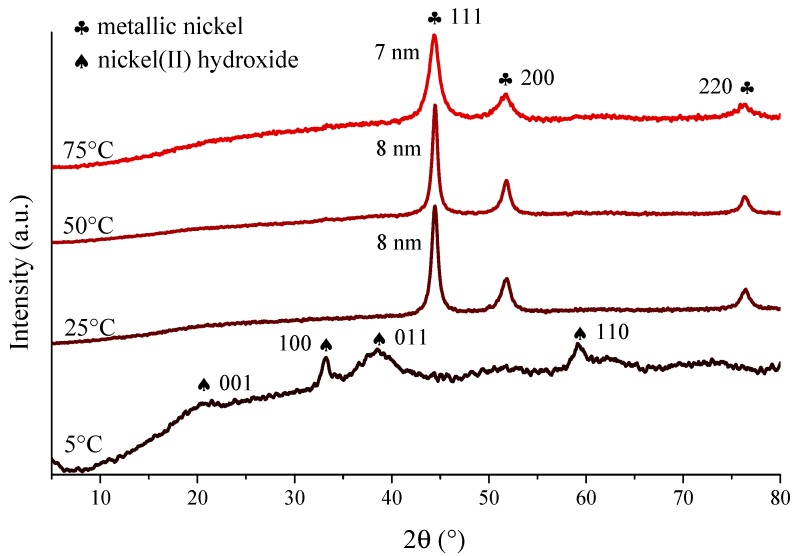
XRD patterns of the nickel nanoparticles prepared by 4 h continuous ultrasound treatment at 30 W output power and varied reaction temperatures.

In the X-ray diffractograms (Figures S1 and S2, Supporting Information), the three reflections with Bragg indices 111, 200 and 220 can be observed indicating the successive evolution of face-centred cubic structure of Ni crystallites (JCPDS#04-0850) in the ultrasonically-aided syntheses as well as those performed with mechanical stirring or without stirring (Figure S2). However, intense baseline rise is seen between 10° and 20° 2*θ* values, a sign for the presence of amorphous phase, for the samples prepared without stirring and low intensity sonication (30 W power and 20% ultrasound emission periodicity). Moreover, significant reduction in the primary crystallite sizes can be observed due to ultrasonic treatments compared to the samples prepared in the non-stirred or the mechanically stirred ways (Table 1). Nevertheless, the estimated sizes only changed slightly on varying the ultrasound operation parameters. On increasing the ultrasound power density, the ultrasound waves with enhanced amplitudes and accelerated emission periodicity resulted in only slight growth of the crystallites, presumably, due to the more vigorous liquid motion and mass transfer induced. In ultrasound treatments, the influence of solvent temperature is generally twofold: the higher temperature means increased vapour pressure inside the cavitation voids and results in less intense collapses, while the formation of cavities got more facile due to the weakening matrix interactions (dipole, van der Waals, hydrogen bonding) between the solvent molecules. To map the exact effect of the ultrasound treatment at various temperature on the formation of nickel nanoparticles detailed investigations were performed between 5 °C and 75 °C (Figure 1). Interestingly, the X-ray powder diffractograms show similar primary crystallite sizes (7–8 nm); this was a remarkably different observation compared to the mechanically stirred cases reported in our previous study [49], where the increasing temperature resulted in the gradual growth in crystallite sizes from 6 nm (at 5 °C) to 14 nm (at 75 °C). On ultrasonic treatment at 25 °C, 50 °C and 75 °C, the diffractograms indicated the presence of metallic nickel, while at 5 °C, the reflections of the *β*-Ni(OH)_2_ intermediate (JCPDS#74-2075) could solely be observed. However, at longer ultrasonic stirring time (6 and 8 h) at this temperature, the most intense 111 reflection of the metallic nickel phase appeared next to the signals of nickel(II) hydroxide (Figure S3).

To understand the effect of the ultrasound treatment on the nucleation and growth steps of the nickel crystals and to find out the reason for the generation of the crystallites in close to constant sizes and for the delayed synthesis of the metallic nickel at 5 °C, detailed knowledge about the formation mechanism of the Ni nanoparticles is indispensable. Nevertheless, the formation mechanism is still not clear in every detail, in spite of the intense research [23,50,51,52]. In the most accepted mechanistic model, the nickel-hydrazine complexes with different hydrazine contents are formed first, then the thermodynamically more stable nickel(II) hydroxide is evolved from the reaction of the hydrazine complex and the alkali additive. The final step is the dissolution of the nanosized nickel hydroxide particles and the reduction of the dissolved Ni(II) ions by the hydrazine molecules. However, the transformation of the solid Ni(OH)_2_ into metallic nickel cannot be excluded, because of the standard redox potential of hydrazine in basic environment (−1.16 eV, N_2_H_4_ + 4 OH^−^ = N_2_ + 4 H_2_O + 4 e^−^) is suitable not only for the reduction of dissolved Ni^2+^ cations (−0.25 eV, Ni^2+^ + 2 e^−^ = Ni^0^), but for the solid Ni(OH)_2_ (−0.72 eV, Ni(OH)_2_ + 2 e^−^ = Ni^0^ + 2 OH^−^) as well. In addition, there are evidences that the alkali additive acts as catalyst [22], and the formation of nickel(III) oxide hydroxide intermediate phase is the precursor of the Ni NPs [53], especially in a reactive environment provided by the propagation of ultrasound in the liquid medium [31].

**Figure 2:**
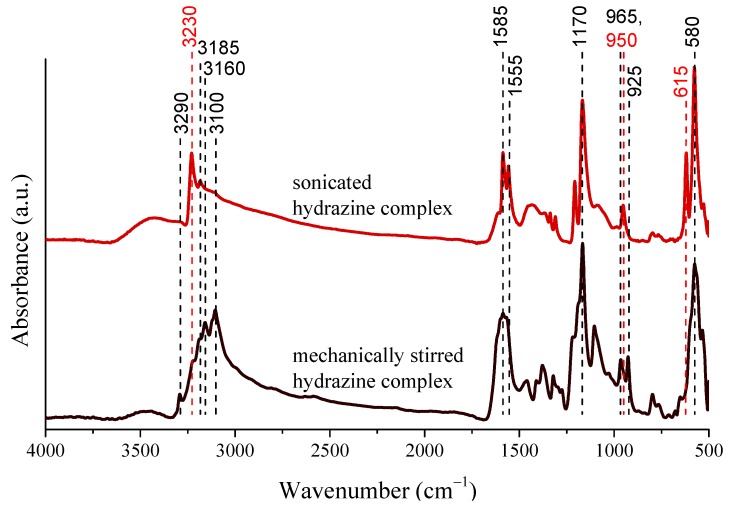
Infrared spectra of the nickel−hydrazine−iodide complexes formed on mechanical stirring or sonication (30 W output power and continuous emission) for 4 h at 75 °C.

Owing to the numerous steps in nanoparticle formation, sonication can affect the formation of Ni NPs in many ways. The hot spots are able to assist in both the formation of unstable and stable nuclei, and the free radicals can further enhance the reduction potential of the synthesis environment [45,54]. Moreover, the rapid (100–200 m/s) liquid microjets penetrate into the voids and their impact on the solid substrates causes their disintegration and turbulent flows in the fluid, hence the solubility of the base, hydrazine complexes/nickel hydroxide/nickel oxide hydroxide can be intensified locally. Considering these factors, possible modifications are expected in the structural properties of the intermediates, therefore our attention was focused at studying the influence of the ultrasound treatment on the solids. For this, X-ray diffractometry and infrared spectroscopy was used. Let us note that the Ni NPs were prepared without additives, the hydroxides were formed in the reaction of the nickel iodide and potassium hydroxide, while the complex particles were prepared through mixing nickel iodide and hydrazine in absolute ethanol under mechanical stirring or ultrasonic treatment. Even though sonication did not result in significant changes in the crystal structure of the nickel hydroxides, the nickel−hydrazine−iodide complexes suffered considerable amorphization (Figure S4). Furthermore, several green dots appeared in the dried solids after sonication, in contrast to the uniformly violet end product obtained with mechanical stirring. The IR spectra of the complexes obtained with mechanical stirring or ultrasonic treatment revealed the characteristic signals of the hydrazine molecules (Figure 2): from above 3000 cm^−1^ the stretching vibrations of the NH_2_ groups and at 1585, 1555 cm^−1^, their bending vibrations are seen. The strong and overlapping adsorption peaks are attributed to their twisting modes around 1170 cm^−1^, while the intense signal at 580 cm^−1^ can be connected to the M−N stretching vibrations [55,56]. On ultrasound treatment, the vibration at 3230 cm^−1^ intensified, the simplified and sharpened twisting mode of the NH_2_ groups, the double peaks at 965 and 925 cm^−1^ connected to the presence of [Ni(N_2_H_4_)_6_] I_2_ and [Ni(N_2_H_4_)_4_] I_2_ vanished, and a new signal appeared at 950 cm^−1^. These observations indicate the sonochemically induced generation of the [Ni(N_2_H_4_)_2_] I_2_ form confirmed by the observed green coloured parts as well [57,58]. Moreover, the appearing peak at 615 cm^−1^ is also originated from the formation of this complex [51].

### 3.2. Analysis of the Aggregation Tendency of the Nanoparticles

**Figure 3:**
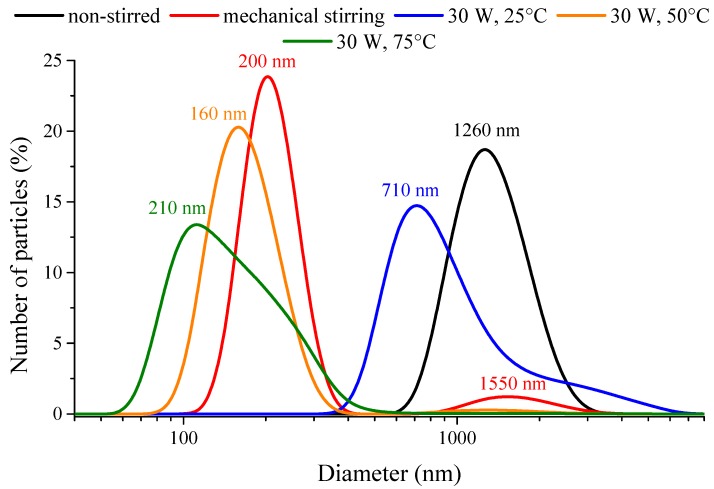
Number-based size distribution curves of the nickel nanoparticles prepared without stirring (25 °C), with mechanical stirring (25 °C) and under ultrasonic treatment (30 W output power, continuous sonication) at various temperatures.

The polydispersity indices (PdI) (from 0.147 to 0.404) obtained from dynamic light scattering measurements attested no strict correlation between the character of the synthesis method used and the heterogeneity of sizes/size distribution of nanoparticle aggregates (Table 1). Nevertheless, the PdI values were largely lower for the ultrasonically prepared samples compared to the mechanical stirred one, but always higher than the non-stirred case. Unimodal size distributions were mostly observed at room temperature, except when mechanical stirring or the highly intense (120 W) continuous ultrasonic treatment was applied. In these instances, bimodal distributions were observed (Figure 3). At 50 °C and 75 °C, the nanoparticles formed secondary, aggregated particles in the range of 50–600 nm with high polydispersity index values of 0.593 and 0.436, respectively. The ultrasound treatment had crucial effects on the aggregation tendency of the nanoparticles: the defragmentation influence of the destroyed cavitation voids with the vigorous mass transfer could overcome the attractive electrostatic and van der Waals forces between the particles. The investigation of the emission periodicity and the output power of the ultrasound treatment proved that the both parameters were essential, by their help, the polydispersity indices could be decreased compared to the mechanically stirred case. The variation of the ultrasound emission towards shorter sonication periods resulted in lower average solvodynamic diameters as did the ultrasonic treatments with amplifying the ultrasound power density; however, the highly intense (120 W) ultrasonic treatment could only result in the lowest solvodynamic diameter of the aggregates.

Five samples were selected for further characterization and catalytic studies. Three of them were prepared by ultrasound treatment and the other two, used as references, by mechanical stirring or without stirring. We intended to study the effects ultrasound periodicity at constant power and of intense (power density 0.085 W/cm^3^) ultrasound treatment on the catalytic properties of nanoparticles, therefore, samples labelled as 30 W – 20%, 30 W – 100% and 120 W – 100% were chosen.

### 3.3. Thermal Properties of the Nickel Nanoparticles

**Figure 4:**
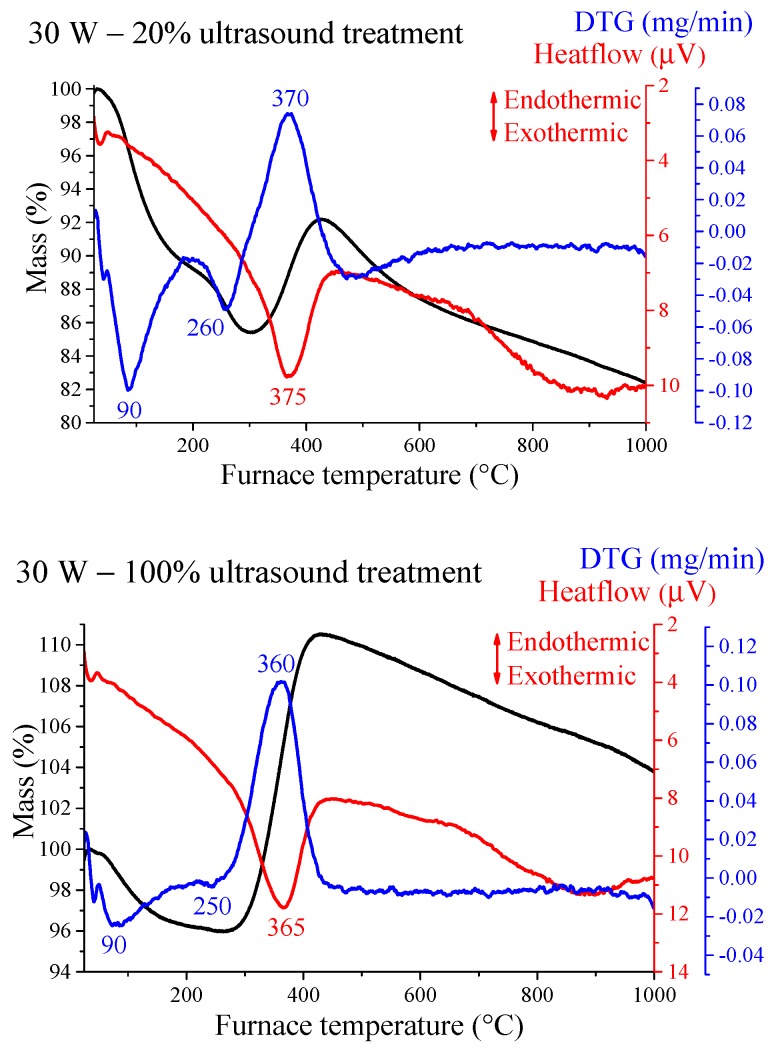
Thermal analysis curves for the sonochemically prepared Ni nanoparticles (NPs) treated at different operating parameters.

First, the thermal behaviour of the selected samples was investigated. Thermal analysis measurements revealed two separate weight losses under 300 °C and a strong mass gain with exothermic peak in the 360–380 °C region for every sample (Figure 4 and Figure S5). The latter signal indicated the oxidation of the nickel particles, the X-ray traces of the residues (Figure S6) displayed the typical reflections of NiO phase (JCPDS#78-0643). Partial oxidation only took place, since the gains in weight were between 12 and 17%, always lower than the amount of the weight increase (27%) corresponding to full oxidation. Moreover, the TG curves indicated gradual mass decrease above 430 °C. These two observations may be originated from the presence of contaminations and/or the continuous formation and decomposition of nickel oxide phases (both from Ni(OH)_2_ and Ni NPs) with significant oxygen deficiency [59,60], and/or the Ni NPs having melting point around 500 °C started to evaporate [61,62].

The first mass loss is connected to the removal of physically adsorbed water molecules from the outer surface, the second one indicates the existence of untransformed and amorphous *β*-Ni(OH)_2_ intermediate residue being largely invisible for the XRD technique. The corresponding mass losses took place in the 245−265 °C temperature range, similarly to those of the as-prepared nickel hydroxide intermediate phase (Figure S5). The quantities and the calculated *β*-Ni(OH)_2_ contents are shown in Table 2. Although the formation of NiO(OH) cannot be ruled out entirely according to the references [63,64], it is presumably minute in amount compared to the extent of nickel hydroxide phase. The highest Ni(OH)_2_ amount was detected for the non-stirred and the 30 W – 20% ultrasonically irradiated samples, where the mass transportations were the lowest delaying the generation of the Ni NPs. The lowest was observed for the mechanical stirred and the 30 W – 100% sonicated samples. The sample irradiated with continuous 120 W output power was in midway indicating that not all featured modifications are induced by intense sonication.

### 3.4. Surface and Porous Properties of the Nickel Nanoparticles

The textural parameters of the nickel nanoparticles were investigated by N_2_ adsorption-desorption measurements, the isotherms were classified as Type IV (according to the IUPAC) with H3 hysteresis (Figure S7). The nanoparticles showed mesoporous structure, their porosity presumably originated from the evolved gaps between the crystallites. The amounts of the total pore volumes and the specific surface areas proved to be diversely dependent on the nature of the applied synthetic method; ultrasound treatment could result in larger average pore diameters expanding the volume of the gaps (Table 2). In general, the specific surface areas/total pore volumes followed directly the amount of the residual *β*-Ni(OH)_2_ resulting in relatively high surface areas and pore volumes (generally ≤100 m^2^/g and ≤0.2 cm^3^/g, respectively) [65,66]. The highest values were calculated for the non-stirred and the 30 W – 20% ultrasonically irradiated, while the lowest ones belong to the mechanically stirred and 30 W – 100% ultrasonically treated samples. The higher surface area of the sample prepared with the most intense ultrasound treatment (120 W − 100%) can be explained by the joint effect of the presence of the Ni(OH)_2_ particles and the enhanced average pore diameter.

### 3.5. CO_2_/NH_3_ Temperate Programmed Desorption (TPD) Studies of the Selected Samples

The application of base is generally required in the cross-coupling reactions, therefore, studying acid-base properties of the nanoparticles was necessary. Table 3 summarizes the calculated values of the total basicity and acidity of the selected Ni NPs originating from the pure metal, the nickel hydroxide residues, the external nickel oxide layers and even the crystal surface defects of the nanoparticles [67], which were potentially multiplied by the ultrasonic cavitation. Note that these TPD investigations somewhat overestimate the number of the acid/base centres due to the formation of the NH_3_–NH_3_ associations [68] and the penetration of the relative small CO_2_ and NH_3_ molecules into the pores hardly accessible for the larger molecules generally used in most organic reactions.

The basicities of the solids were close to what were indirectly determined by the amount of Ni(OH)_2_ residues. The 30 W – 20% and the 120W – 100% samples proved to be the most basic, while the mechanically stirred and the 30 W – 100% samples displayed lower basicities. The CO_2_-TPD profiles showed two peaks under 300 °C; the first at ~90 °C originating from the weak dative bonds of the NiO and the CO_2_ species, while the second, with maxima between 155–175 °C can be assigned to stronger adsorption interaction [69].

The total acidities of the solids were also dependent on the NiO content. The NH_3_-TPD traces revealed that the 30 W − 100% and the mechanically stirred samples had the weakest (150 °C) as well as the lowest number of acidic centres, while the other samples had well-observable desorption maxima around 180 °C derived from desorption of hydrogen-bonded NH_3_ molecules [70]. For the mechanically stirred and the 30 W − 100% ultrasonically prepared samples, peaks with maximum at 330 °C and 370 °C, respectively, were observed, probably due to the desorption of terminally bound NH_3_ [71].

Although these measurements cannot distinguish the Brønsted and Lewis acid sites; however, because of the lack of mobile protons we can safely state that they are Lewis acid sites. Overall, it is seen that the less acidic solids were the non-stirred and the mechanically stirred samples, while the 30 W − 100% sample had significantly more moderate acidic sites. For these two last samples, a large difference can be observed; the total acidity of the sonically prepared nanoparticles was ten times higher, however, their specific surface areas (even if we note the change of the textural parameters during the dehydration of the Ni(OH)_2_), nickel hydroxide contents were largely similar. It may be connected to the smaller total pore volume of the mechanically stirred ones meaning lower accessible pores/space for the adsorption of NH_3_ molecules. Another possible explanation can be the ultrasound induced modification in the number of the Ni step-like and terrace-like sites due to the electron donation character of the negatively charged terraces and electron accepting capability of the positive steps [72]. This interpretation could be well applied to translate the lower basicity of the ultrasonically (30 W – 100%) prepared solids compared to the mechanically stirred ones.

### 3.6. The selected Materials Studied by Scanning Electron Microscopy

**Figure 5:**
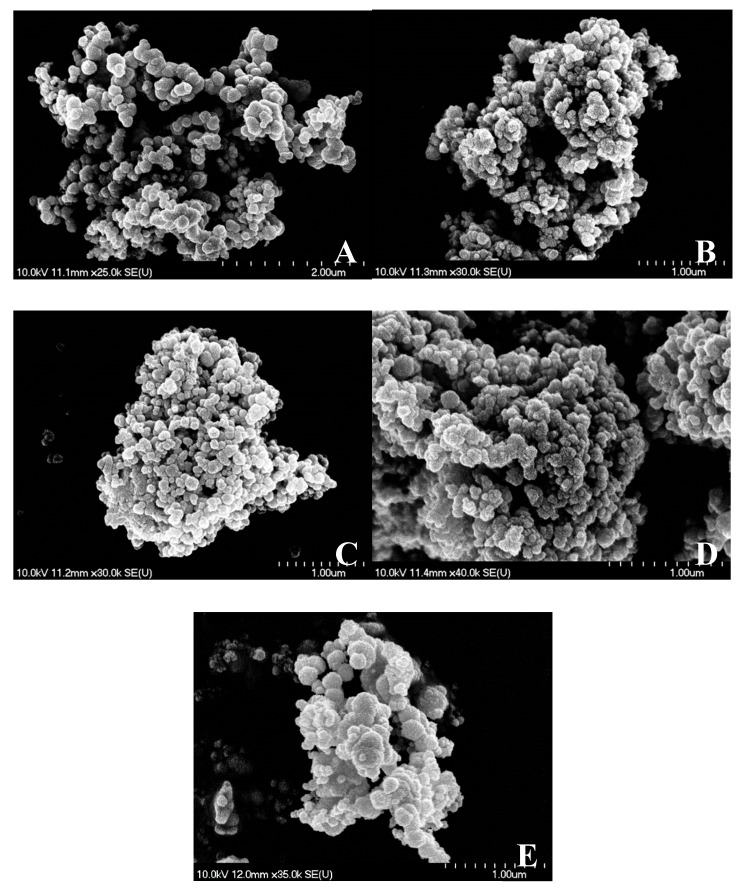
Scanning electron microscopy images of the selected NiNPs (**A**: non-stirred, **B**: mechanically stirred, **C**: 30 W – 20%, **D**: 30 W – 100%, **E**: 120 W – 100% ultrasonically treated samples).

Scanning electron microscopy measurements revealed the largely uniform morphology of the nanoparticles. Close to spherical particles were observed in the size range of 200–400 nm merged into larger (>1 μm) aggregates independently from the method of synthesis (Figure 5). However, continuous ultrasound treatment at 120 W output power could enhance slightly the formation of the larger and less symmetric spheres, presumably due to the more intense mass transfer and deforming/frittering effect related to the continuously forming and eliminating cavities. The EDX probes showed no sign of iodine atoms as direct evidence of the complete transformation of the starting material; however, oxygen atoms were seen confirming the presence of nickel hydroxide intermediate. Potassium were found in negligible amount (25−30:1 Ni:K molar ratio) as the residue of the alkali additive applied (Figure S8).

## 4. Catalytic Application of the Ni NPs in the Suzuki-Miyaura Cross-Coupling Reaction

**Scheme 1:**
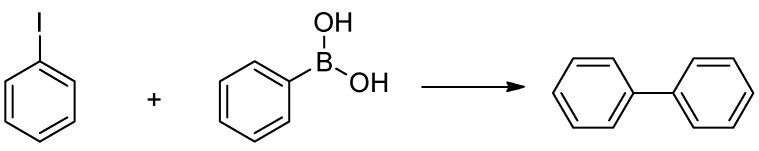
The Suzuki-Miyaura cross-coupling test reaction between the iodobenzene and phenylboronic acid.

**Figure 6:**
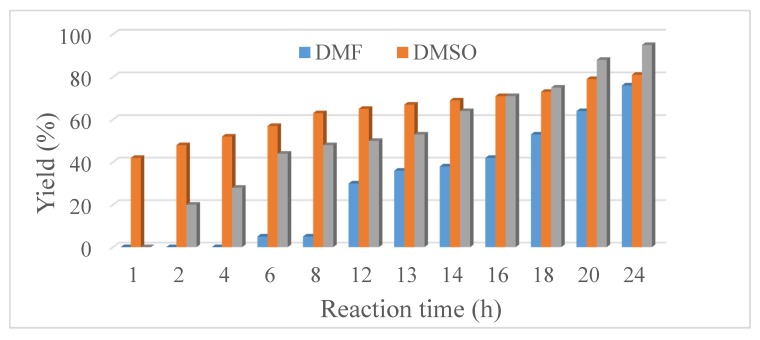
The biphenyl yield in the reaction of iodobenzene and phenylboronic acid in different solvents using Ni catalyst prepared with continuous ultrasound treatment (30 W output power) and at reflux temperature.

In recent decades, the impact of the Suzuki-Miyaura cross-coupling (SMC) reaction on academic and industrial research has become immense. The main advantages of these reactions are the mild reaction conditions and the high tolerance toward functional groups. Hence, the SMC reaction is often applied in the large-scale synthesis of fine chemicals and pharmaceuticals [73]. Originally, expensive palladium- [74,75] and nickel-based complexes [76,77] were used. In this work, our aim was to use the economically more accessible pure nickel metal in the synthesis of biphenyl product (Scheme 1) enhancing its activity by ultrasonic treatment. For scouting experiments the 30 W – 100% sample was used, and the investigations were started with varying the reaction time and the solvents (Figure 6). The solvents chosen were toluene, dimethyl sulfoxide (DMSO) and dimethyl formamide (DMF) with water content of 20% v/v in order to aid the dissolution of the K_2_CO_3_ base. In DMSO, the yield after 24 h was 81%. Interestingly, the biphenyl product only appeared after a 1 h induction period in toluene; however, the yield after 24 h was 95%. In DMF, the yield after 24 h was only 76%.

**Figure 7:**
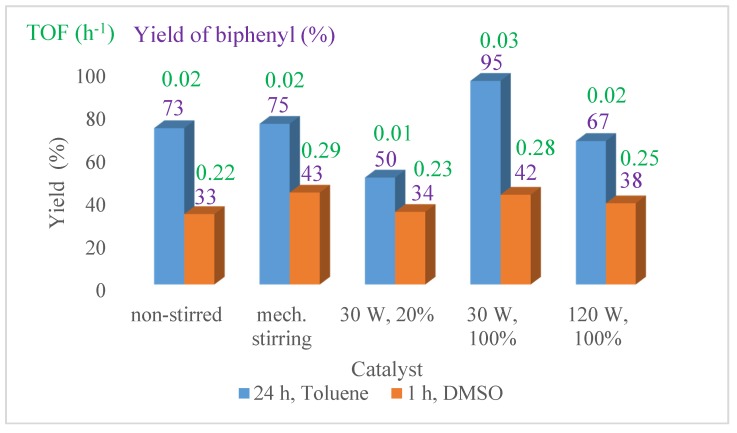
The biphenyl yield in the reaction of iodobenzene and phenylboronic acid in toluene after 24 h (blue) and in DMSO after 1 h (red) with the selected NiNPs samples at reflux temperature.

Then, for testing the five selected NiNP samples, DMSO and toluene solvents were used, and sampling was performed after 1 h and 24 h reaction time, respectively (Figure 7). The sampling times were chosen according to the calculated turn over frequency (TOF = (mol of product/mol of catalyst)/reaction time) values (Figure S9); in DMSO, the nanoparticles showed the highest activity in the first hour of the cross-coupling reactions, while it needed more than 20 h in the DMF. The toluene was selected due to the close 100% yield of biphenyl synthesis, however, the nanoparticles showed the lowest activity here.

The nanoparticles prepared with continuous ultrasound treatment at 30 W output power proved to be the most active catalyst in toluene, interestingly, the other two sonicated samples (30 W – 20%, 120 W – 100%) showed significantly more modest performance, the yields were even lower than those over the mechanically stirred or the non-stirred materials.

Similarly, in DMSO, the yield and the TOF values were the highest for the mechanically stirred and the 30 W – 100% ultrasonically synthesised NiNPs.

Although many variables can influence the performance of the catalysts, it is to be noted that the 30 W – 100% catalyst has higher Lewis acidity and it performs significantly better in the 24 h reactions compared than the other catalysts. It is also known that the oxidative addition steps of the cross-coupling reactions are successfully promoted by Lewis acid additives [78,79,80,81]. Therefore, we propose that the surface Lewis centres, located in atomic scale close vicinity of the metallic nickel atoms, aid the formation of the organonickel species through weakening the aryl carbon–iodine bonds [79,82].

As far as recycling properties are concerned, the used 30 W – 100% ultrasonically prepared catalyst was investigated by X-ray diffractometry after the first 24 h reactions in both toluene and DMSO. Originally, the NiNPs had the face-centred (fcc) cubic structure identified by the corresponding reflections. In DMF, this was partially transformed to hexagonal close-packed (hcp) structure (JCPDS#45-1027) and nickel oxide (JCPDS#78-0643) (Figure S10).

The reusability of the nanoparticles prepared with mechanical stirring and the 30 W – 100% NiNPs synthesized with ultrasonic treatment were further studied in DMSO and toluene, but the reaction time was decreased to 1 h when DMSO was the solvent. In toluene, the ultrasonically synthesized NPs gave 71% yield, while the mechanical stirred ones produced 58% yield in the first reuse experiment, in DMSO, the yields were 35% and 23%, respectively. The reason of the reduced yields is probably in direct correlation with the revealed structural modifications (Figure S11). The NiNPs prepared with mechanical stirring maintained their original crystal framework in toluene, but with significant amorphization, while unidentified reflections were found beside the signals of the fcc NiNPs in DMSO. The ultrasonically prepared samples displayed different behaviour; in toluene, a mixture of NiO, fcc and hcp NiNPs was recorded, while in DMSO, NiS (JCPDS#12-0041) phase was solely observed. The EDX analysis revealed the presence of sulphur in the both catalysts after the reactions performed in DMSO solvent indicating chemical reaction between the nickel nanoparticles and the solvent molecules (Figures S12 and S13).

## 5. Conclusions

Ultrasound treatment was successfully utilized to influence the hydrazine reduction synthesis method of the nickel nanoparticles. The sonically induced intermediate complexes for the variously treated materials could be identified. Sonication was able to maintain an average primary crystallite size (7–8 nm) independently from the applied temperature, while the use of intense and shorter sonication periods could reduce the solvodynamic diameters of the secondary, aggregated particles from 710 nm to 190 nm in the absence of any surfactant. The highest acidity and catalytic activity were measured for the nanoparticles prepared by mild (30 W output power) and continuous ultrasound treatment.

The work is dedicated to Professor Grzegorz Mlostoń on the occasion of his 70th birthday.

## Figures and Tables

**Table 1:** Acoustic energy, particle and dispersion dimensions of the nickel nanoparticles prepared at 25 °C.

Ni NPs Samples	Ultrasound Power Density (W/cm^3^) ^a^	Primary Particle Size (nm)	Predominant Solvodynamic Diameter (nm)	Poly-Dispersity Index	Type of Size Distribution
non-stirred	−	12	1262	0.147	Unimodal
Mechanically stirred	−	14	202	0.368	Bimodal
ultrasonic treatment:					
30 W – 20% ^b^	0.007	7	255	0.277	Unimodal
30 W – 40%	0.01	7	396	0.199	Unimodal
30 W – 60%	0.013	7	342	0.288	Unimodal
30 W – 80%	0.017	8	396	0.230	Unimodal
30 W – 100%	0.023	8	712	0.170	Unimodal
60 W – 100%	0.041	8	825	0.198	Unimodal
90 W – 100%	0.057	9	530	0.226	Unimodal
120 W – 100%	0.085	10	190	0.404	Bimodal

^a^ Calculated to 5 cm^3^ sample volume. ^b^ The percentages mean the emission periodicity of the sonication, for instance, at 40%, the device was inactive in the three fifth of the pulse and the sonication period was two fifth of the active time, while at 100%, the ultrasonic homogenizer operated continuously.

**Table 2:** The results of the thermogravimetric (TG) analysis and the N_2_ adsorption-desorption measurements of the nickel nanoparticles.

Ni NP Samples	TG Second Mass Loss (%)	*β*-Ni(OH)_2_ Content (m/m%)	Specific Surface Area (m^2^/g)	Total Pore Volume (cm^3^/g)	Average Pore Diameter (Å)
non-stirred	2.2	11.3	28.1	0.040	35.4
mechanical stirring	0.3	1.5	21.9	0.027	35.8
30 W – 20% ^a^	3.7	19.0	39.5	0.054	38.8
30 W – 100%	0.3	1.5	20.4	0.043	36.2
120 W – 100%	1.0	5.1	28.6	0.036	38.8

^a^ The percentages mean the emission periodicity of the ultrasound treatment, for instance, at 20%, the ultrasonic homogenizer was inactive in the four fifth of the pulse and the sonication period was one fifth of the active time, while at 100%, the device worked continuously.

**Table 3:** Summary of the temperature-programmed desorption (TPD) results for the selected materials.

Ni NP Samples	Total Basicity (mmol CO_2_/g)	Temperature of Peak Maxima (°C)	Total Acidity (mmol NH_3_/g)	Temperature of Peak Maxima (°C)
non-stirred	0.051	90 and 165	0.021	185 and –
mechanically stirred	0.046	90 and 160	0.025	150 and 330
30 W – 20%	0.054	95 and 155	0.105	175 and –
30 W – 100%	0.032	90 and 175	0.257	150 and 370
120 W – 100%	0.088	90 and 165	0.054	190 and –

## Supplementary Materials

The following are available online at https://www.mdpi.com/2079-4991/10/4/632/s1, Figure S1: XRD patterns of the nickel nanoparticles prepared under ultrasound treatment with various ultrasound emission periodicities (duration of treatment: 4 h, temperature of treatment: 25 °C); Figure S2: XRD patterns of the nickel nanoparticles prepared with mechanical stirring, without stirring or under ultrasonic treatment at various output power values (duration of treatment: 4 h, temperature of treatment: 25 °C); Figure S3: XRD patterns of the solid materials formed on ultrasound treatment (30 W − 100%) at 5 °C with 4 h, 6 h or 8 h treatments or without stirring at 5 °C after 4 h; Figure S4: XRD patterns of the nickel hydroxide and complex intermediates obtained under mechanical stirring or sonication (30 W − 100%) at 75 °C, after 4 h treatment; Figure S5: Thermogravimetric curves of the β-Ni(OH)2 and the NiNPs prepared without stirring, with mechanical stirring or under ultrasound treatment (120 W − 100%); Figure S6: X-ray patterns of the thermogravimetric residues of the nanoparticles; Figure S7: N2 adsorption-desorption (left) and the pore size distribution (right) curves of the selected nickel nanoparticles; Figure S8: Energy dispersive X-ray analysis spectrum of NiNPs prepared at room temperature with ultrasound treatment of 30 W output power and 20% emission periodicity (signals of carbon, aluminum and phosphorous are from the adhesive tape/sample holder); Figure S9: Evolution of the turn over frequency (TOF) values of the ultrasonically synthesised nanoparticles (30W, continuous sonication) during the cross-coupling reaction in DMF, DMSO and toluene solvents; Figure S10: XRD patterns of the used nickel nanoparticle catalyst (30 W − 100%) after the first 24 h run in various media (fcc—face-centered, hcp—hexagonal close-packed); Figure S11: XRD patterns of NiNPs prepared by 30 W − 100% ultrasound treatment and mechanical stirring after the repeated run in toluene and DMSO solvents (fcc—face-centered, hcp—hexagonal close-packed); Figure S12: Energy dispersive X-ray analysis spectrum of NiNPs prepared with ultrasound treatment (30 W − 100%), after using it as catalyst in DMSO solvent (signals of carbon and oxygen originate from the adhesive tape/sample holder, the Ni:S molar ratio ~1:1); Figure S13: Energy dispersive X-ray analysis spectrum of NiNPs synthesized with mechanical stirring, after using it as catalyst in DMSO solvent (signs of the carbon, aluminium and the oxygen could originate from the adhesive tape/sample holder, the Ni:S molar ratio ~1:1).
