# Supplementary material for: Ultrasound-Assisted Hydrazine Reduction Method for the Preparation of Nickel Nanoparticles, Physicochemical Characterization and Catalytic Application in Suzuki-Miyaura Cross-Coupling Reaction
Source: Nanomaterials (Basel). 2020 Mar 28;10(4):632. doi: 10.3390/nano10040632 (PMC7221950; doi:10.3390/nano10040632)
Supplement: Supplementary file 1 [file nanomaterials-10-00632-s001.pdf]

Supplementary Materials: Ultrasound-Assisted Hydrazine Reduction Method for the Preparation of Nickel Nanoparticles, Physicochemical Characterization and Catalytic Application in Suzuki-Miyaura Cross-Coupling Reaction

Adél Anna Ádám [1,2], Márton Szabados [1,2], Gábor Varga [1,2], Ádám Papp [2], Katalin Musza [1,2], Zoltán Kónya [3,4], Ákos Kukovecz [3], Pál Sipos [2,5] and István Pálinkó [1,2,*]

[1] Department of Organic Chemistry, University of Szeged, Dóm tér 8, Szeged, H-6720 Hungary, leedaa@hotmail.com (A.A.A.); szabados12m@gmail.com (M.S.); gaborvarga1988@gmail.com (G.V.); musza.katalin@chem.u-szeged.hu (K.M.).

[2] Material and Solution Structure Research Group, and Interdisciplinary Excellence Centre, Institute of Chemistry, University of Szeged, Aradi Vértanúk tere 1, Szeged, H-6720 Hungary, papy97@gmail.com (A.P.); sipos@chem.u-szeged.hu (P.S.).

[3] Department of Applied and Environmental Chemistry, University of Szeged, Rerrich B. tér 1, Szeged, H-6720 Hungary, konya@chem.u-szeged.hu (Z.K.); kakos@chem.u-szeged.hu (A.K.).

[4] MTA-SZTE Reaction Kinetics and Surface Chemistry Research Group, Rerrich B tér 1, Szeged, H-6720 Hungary

[5] Department of Inorganic and Analytical Chemistry, University of Szeged, Dóm tér 7, Szeged, H-6720 Hungary

* Correspondence: palinko@chem.u-szeged.hu

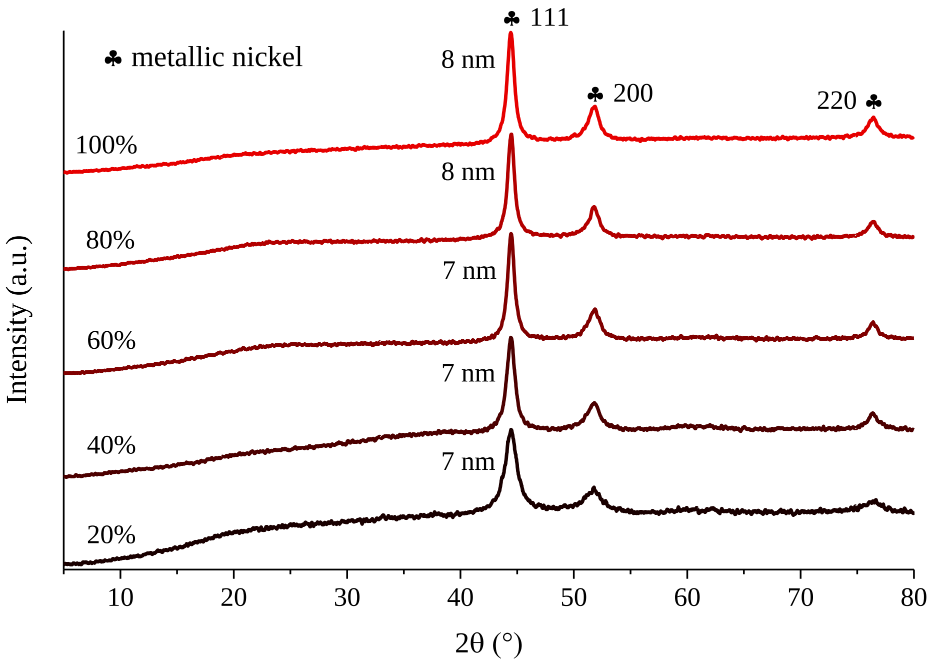

Figure S1. XRD patterns of the nickel nanoparticles prepared under ultrasound treatment with various ultrasound emission periodicities (duration of treatment: 4 h, temperature of treatment: 25 °C).

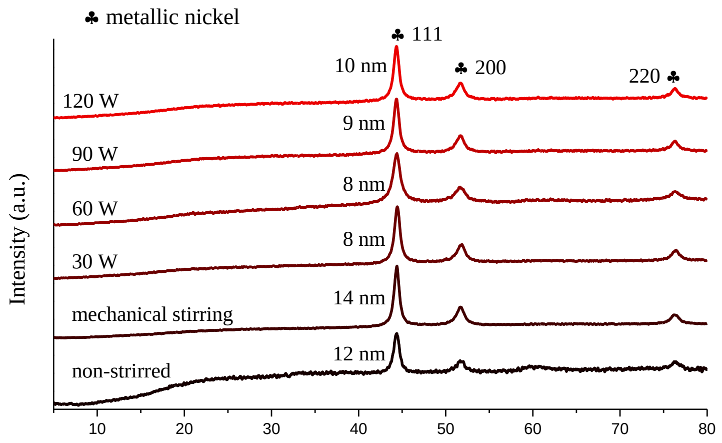

Figure S2. XRD patterns of the nickel nanoparticles prepared with mechanical stirring, without stirring or under ultrasonic treatment at various output power values (duration of treatment: 4 h, temperature of treatment: 25 °C).

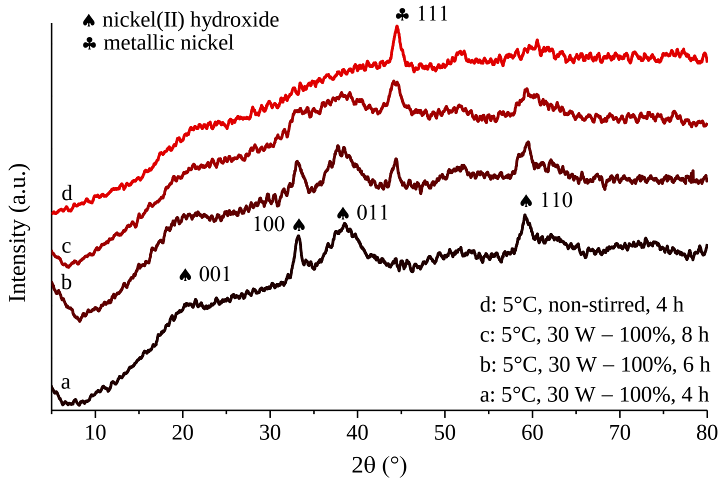

Figure S3. XRD patterns of the solid materials formed on ultrasound treatment (30 W – 100%) at 5 °C with 4 h, 6 h or 8 h treatments or without stirring at 5 °C after 4 h.

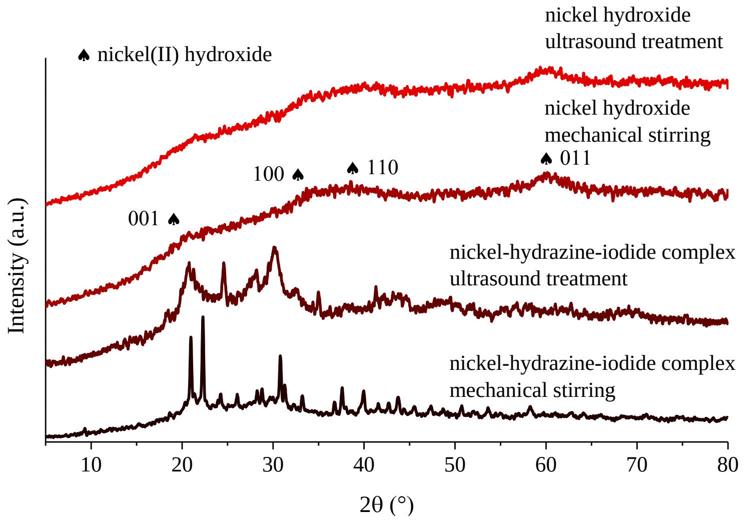

Figure S4. XRD patterns of the nickel hydroxide and complex intermediates obtained under mechanical stirring or sonication (30 W – 100%) at 75 °C, after 4 h treatment.

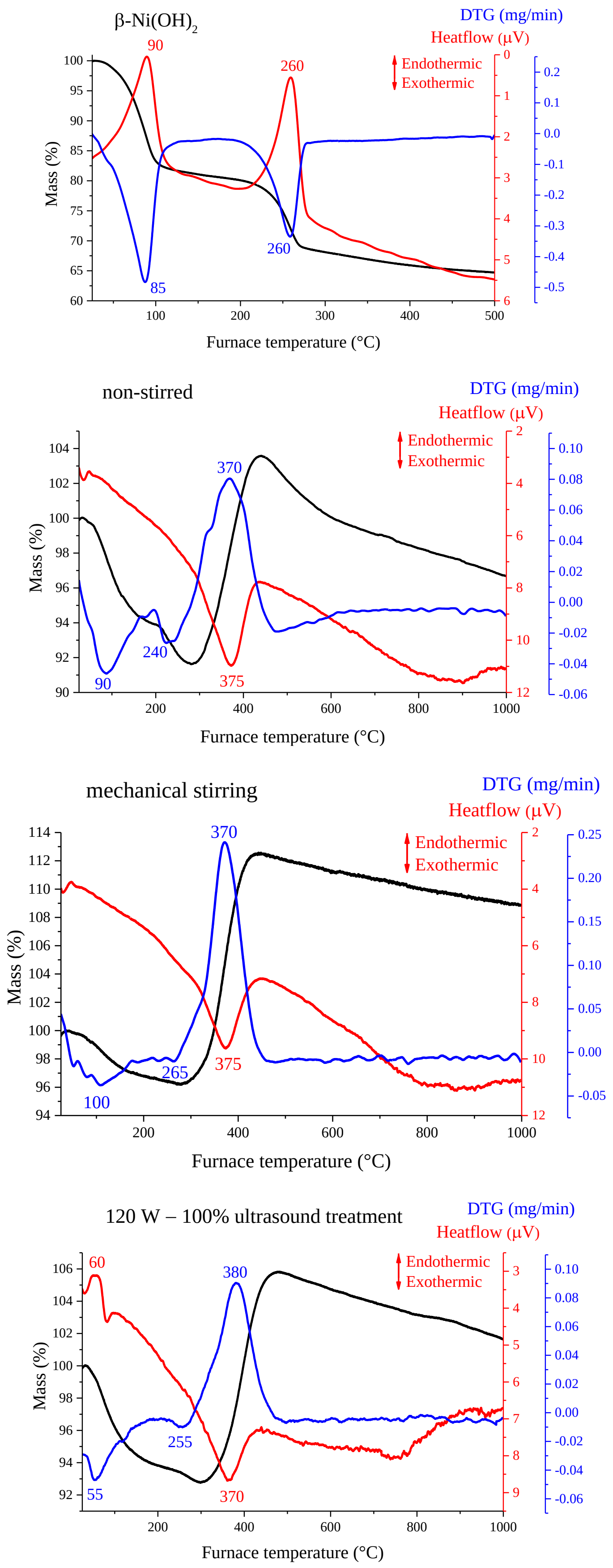

Figure S5. Thermogravimetric curves of the β-$Ni(OH)_2$ and the NiNPs prepared without stirring, with mechanical stirring or under ultrasound treatment (120 W – 100%).

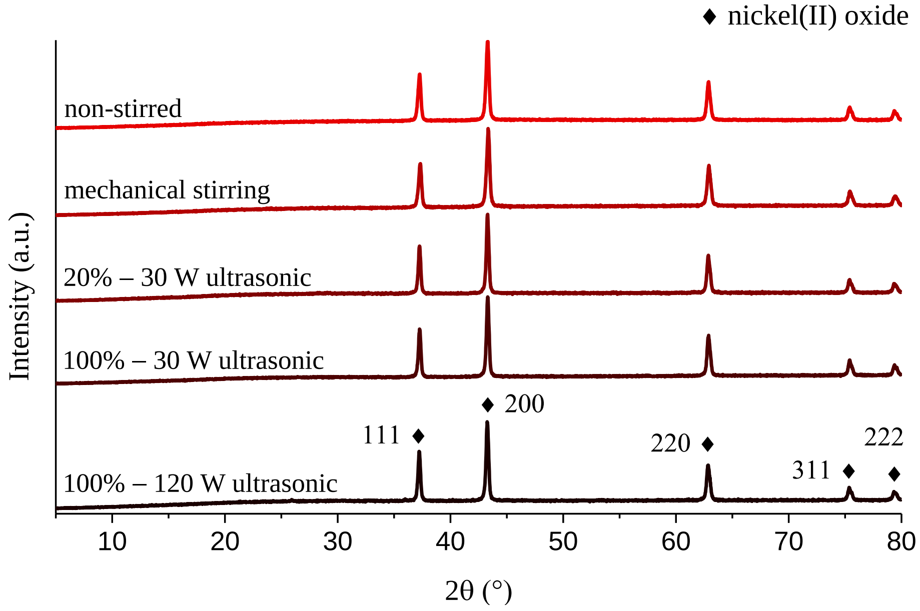

Figure S6. X-ray patterns of the thermogravimetric residues of the nanoparticles.

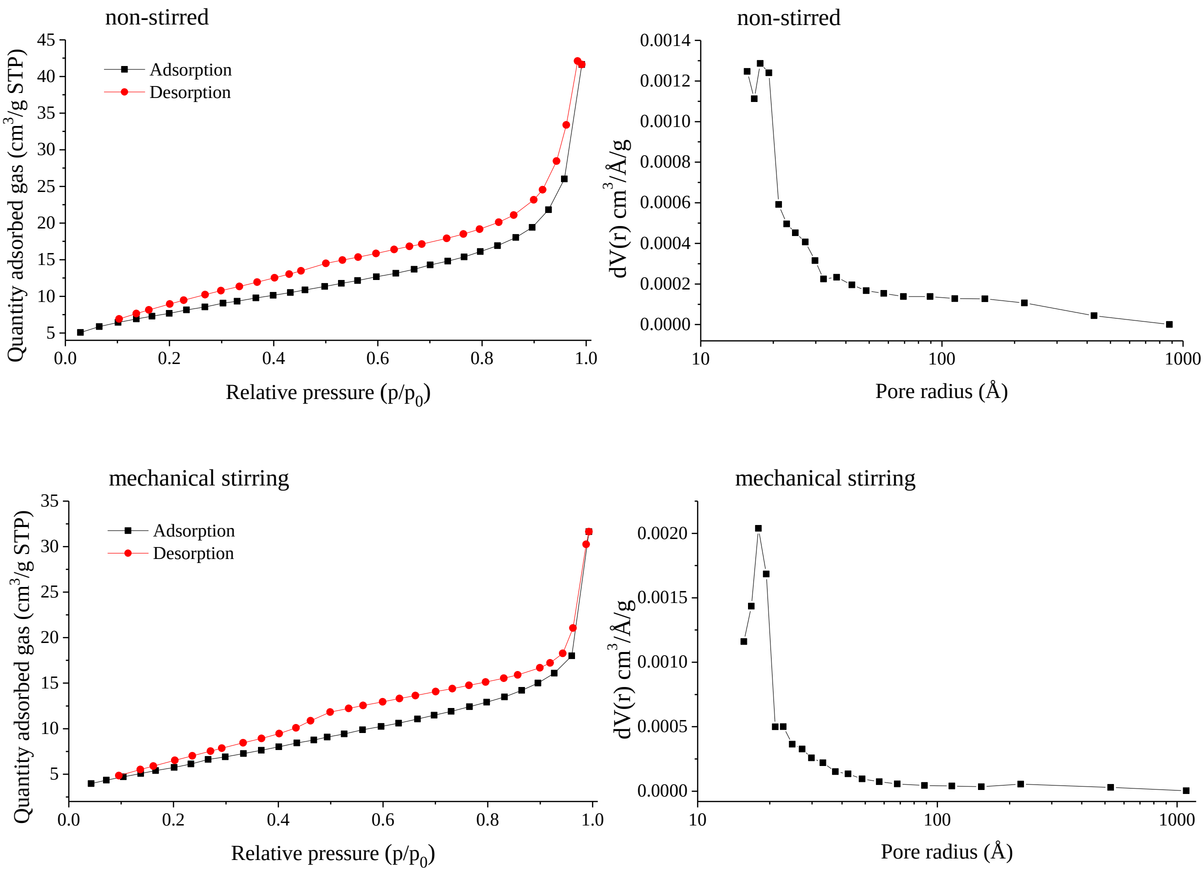

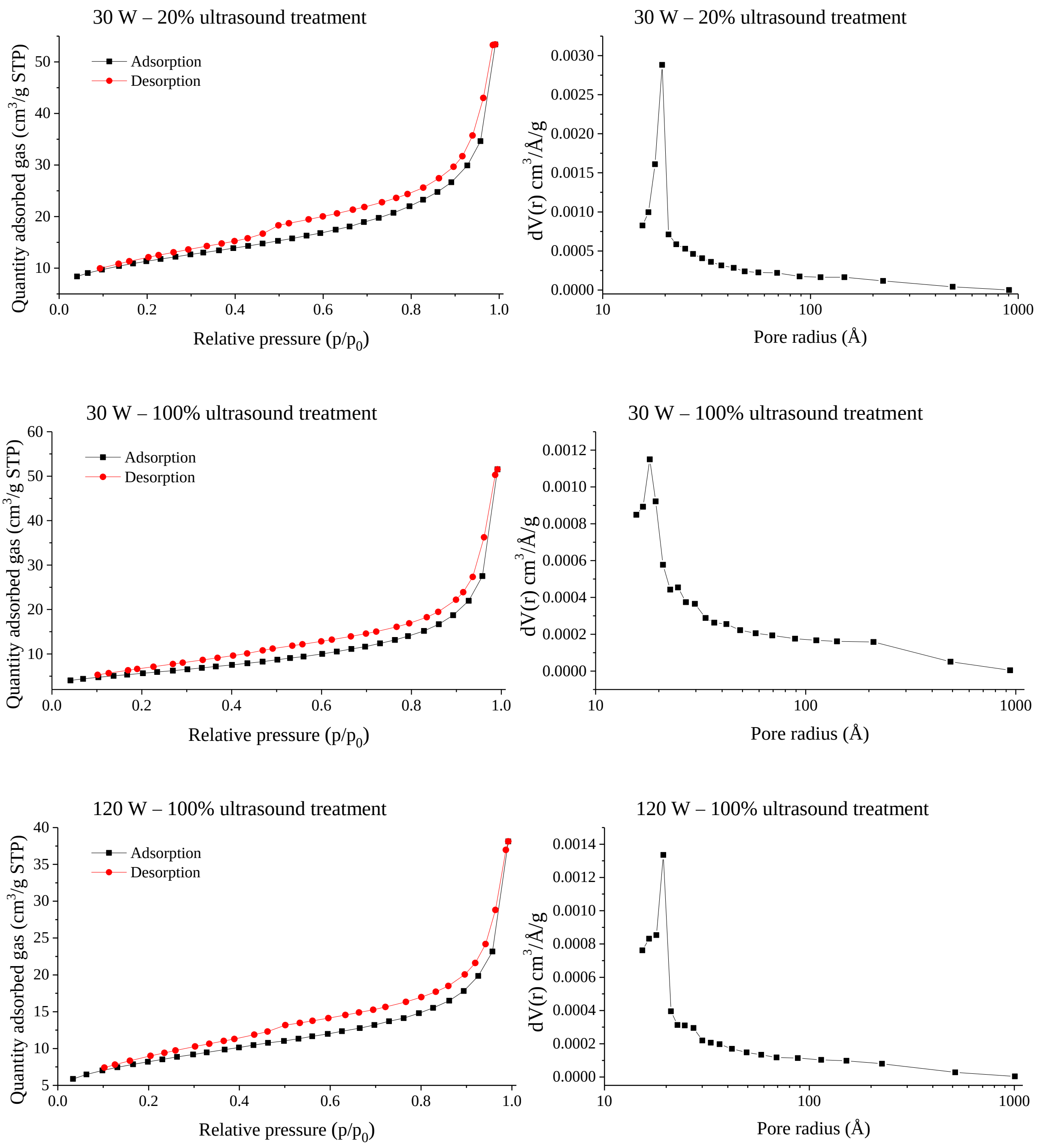

Figure S7. N_2 adsorption-desorption (left) and the pore size distribution (right) curves of the selected nickel nanoparticles.

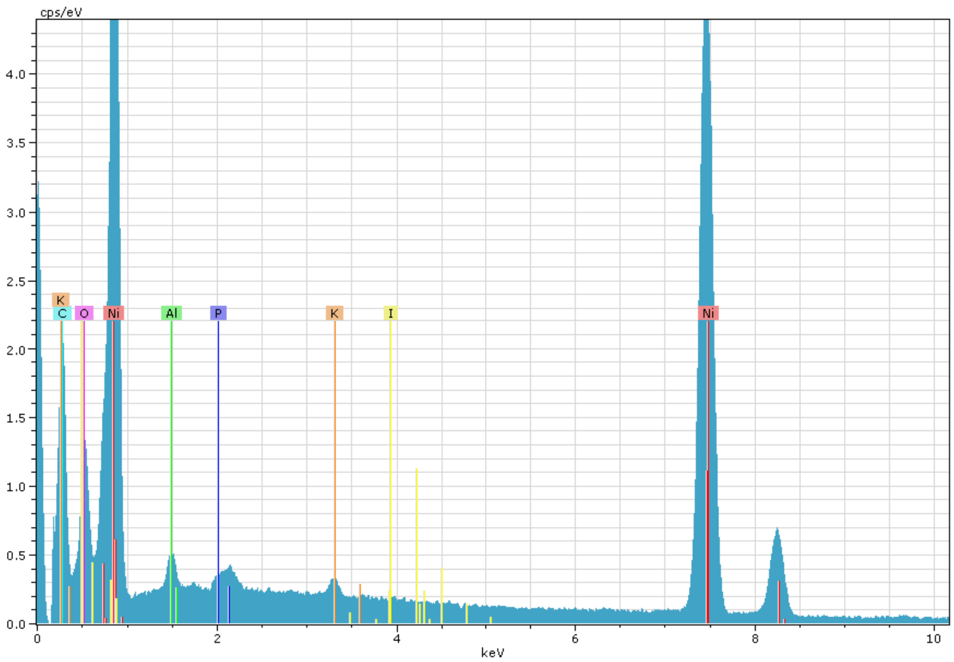

Figure S8. Energy dispersive X-ray analysis spectrum of NiNPs prepared at room temperature with ultrasound treatment of 30 W output power and 20% emission periodicity (signals of carbon, aluminum and phosphorous are from the adhesive tape/sample holder).

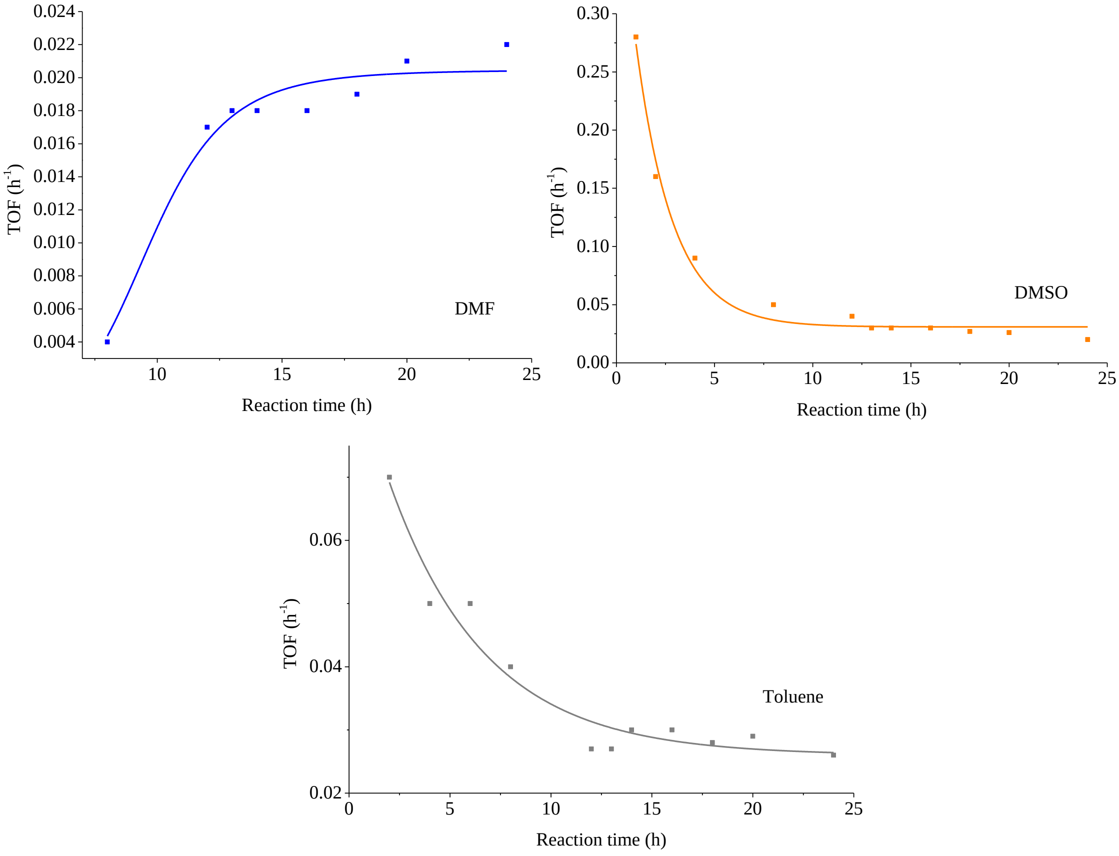

Figure S9. Evolution of the turn over frequency (TOF) values of the ultrasonically synthesised nanoparticles (30 W, continuous sonication) during the cross-coupling reaction in DMF, DMSO and toluene solvents.

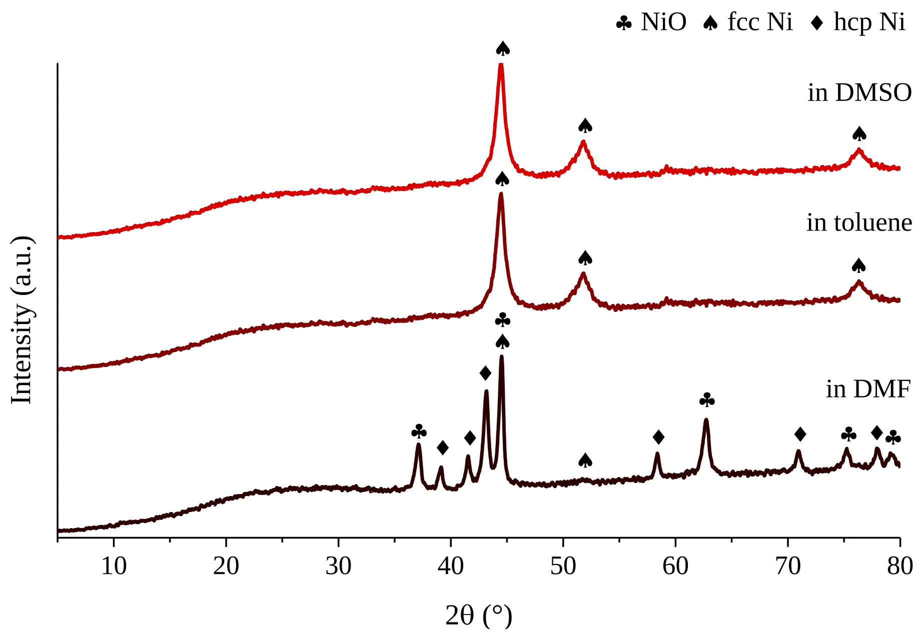

Figure S10. XRD patterns of the used nickel nanoparticle catalyst (30 W – 100%) after the first 24 h run in various media (fcc—face-centered, hcp—hexagonal close-packed).

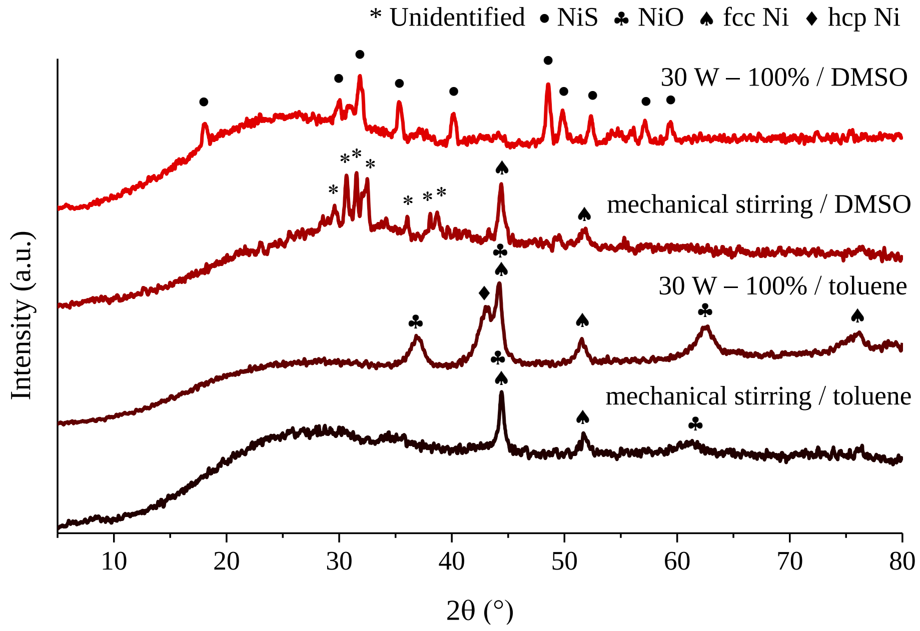

Figure S11. XRD patterns of NiNPs prepared by 30 W – 100% ultrasound treatment and mechanical stirring after the repeated run in toluene and DMSO solvents (fcc—face-centered, hcp—hexagonal close-packed).

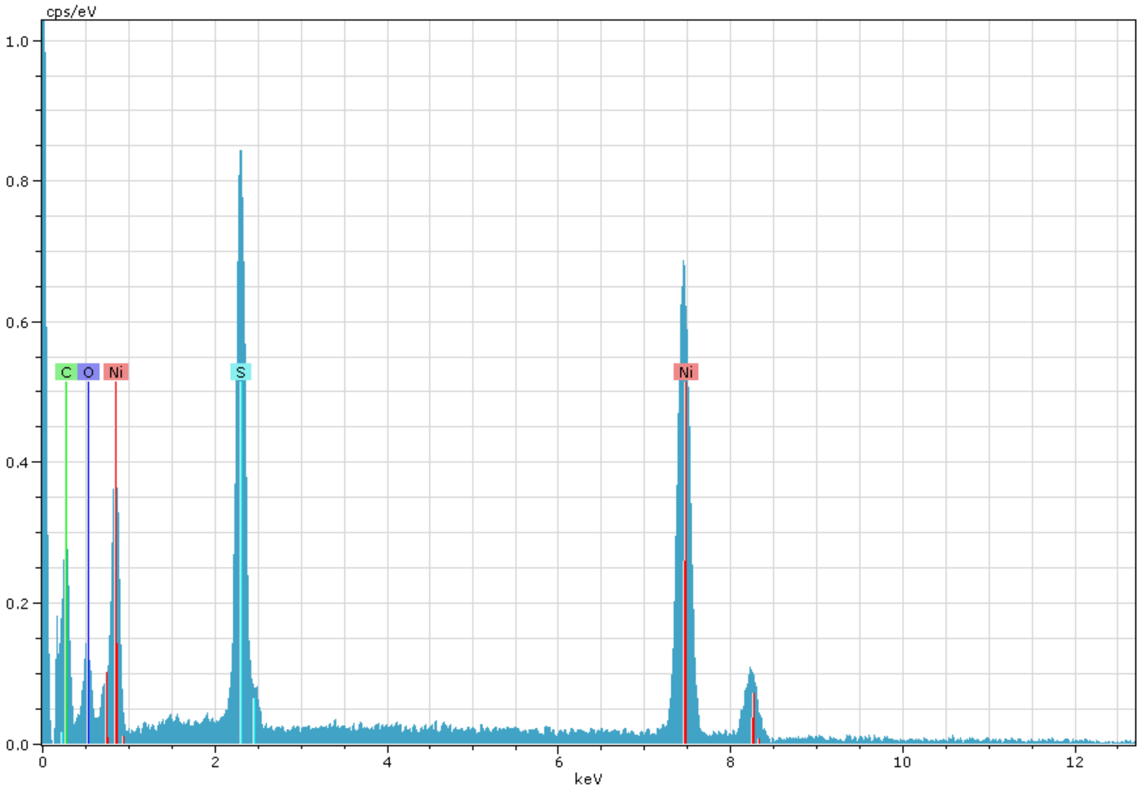

Figure S12. Energy dispersive X-ray analysis spectrum of NiNPs prepared with ultrasound treatment (30 W − 100%), after using it as catalyst in DMSO solvent (signals of carbon and oxygen originate from the adhesive tape/sample holder, the Ni:S molar ratio ~1:1).

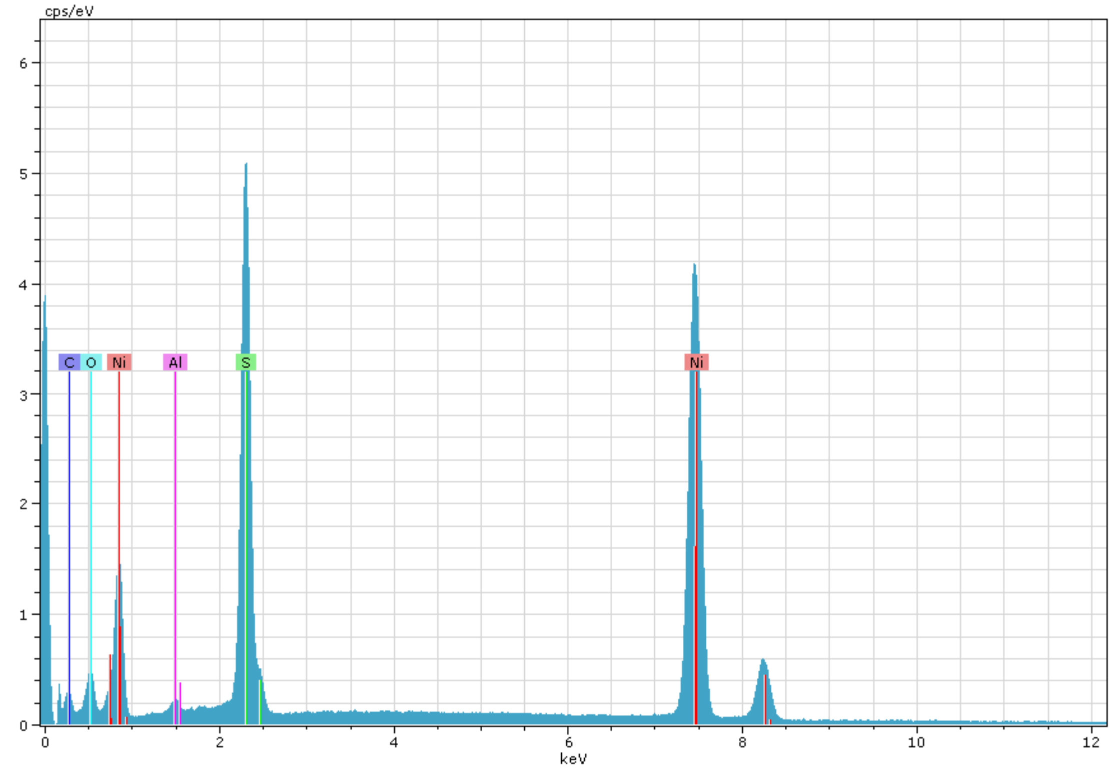

Figure S13. Energy dispersive X-ray analysis spectrum of NiNPs synthesized with mechanical stirring, after using it as catalyst in DMSO solvent (signs of the carbon, aluminium and the oxygen could originate from the adhesive tape/sample holder, the Ni:S molar ratio ~1:1).
